# Evaluation of Demographics and Management of Rectal Cancer by Age Group: A Retrospective Propensity Matched Cohort Analysis of the National Cancer Database

**DOI:** 10.7759/cureus.19412

**Published:** 2021-11-09

**Authors:** Jonathan Bliggenstorfer, Katherine Bingmer, Asya Ofshteyn, Anuja L Sarode, Meridith Ginesi, Sharon L Stein, Emily Steinhagen

**Affiliations:** 1 Department of Surgery, University Hospitals Research in Surgical Outcomes and Effectiveness (UH RISES), Cleveland, USA

**Keywords:** rectal cancer, disparities, early age of onset, oncology, national cancer database

## Abstract

Background

Data suggests there are demographic and biological differences in colon cancer between young and typical-onset patients. However, it is unclear if these differences persist in rectal cancer patients, exclusive of colon cancer. This is a retrospective review of a large national database to evaluate age-based differences in demographics, tumor features, and treatment among patients with rectal adenocarcinoma.

Methods

The National Cancer Database from 2004-2014 was queried for rectal adenocarcinoma. Patients were grouped by age at diagnosis: early-onset, defined as <40 years, mid-onset 40-49, and late-onset ≥50. Propensity matching controlled for demographic variation among cohorts. Pairwise Chi-square with Bonferroni correction was used for analysis.

Results

Thirty thousand nine hundred seventy-eight patients were included: 1,249 (4%) early-onset, 4,156 (13%) middle-onset, and 25,573 (83%) late-onset. Significant differences existed between all three cohorts in nearly all demographic and pathologic metrics. Control for demographic variation revealed early-onset and middle-onset cohorts differed only with respect to the stage at presentation, while early-onset and late-onset cohorts differed more significantly on the basis of stage, histology, and oncologic management.

Conclusion

The demographic differences observed demonstrate that patients under 50 should not be considered one cohort. Propensity matching led to a decrease in tumor trait differences among cohorts, suggesting that demographics other than age drive variation in tumor biology. Young patients received more aggressive management, implying the presence of an age bias. Age-based screening is likely insufficient and may exclude the rising proportion of young patients at risk for disease, while age-based management may lead to under- or overtreatment of patients at either end of the age spectrum.

## Introduction

A rise in the incidence of colorectal cancer (CRC) has been noted among patients under 50 years of age, despite a decline in the overall incidence of CRC in recent decades [1,2]. This observation has triggered research to improve our understanding of this trend, with literature demonstrating clinicodemographic differences between younger and older patients with CRC. It has been well-documented that younger patients are more likely to be minorities, present with left-sided colon or rectal tumors, have advanced American Joint Committee on Cancer (AJCC) stage, and have unfavorable histology with higher histologic grades [3-6].

While a great deal of research has pointed to the differences between younger and older CRC populations, these studies often group colon and rectal cancers as a single disease process [7]. Evidence suggests colon and rectal cancers should not be evaluated together, given differing embryologic origin, metastatic patterns, and drug targets [8]. Therefore, analyzing colon and rectal cancer together may cloud differences between younger and typical-onset patient populations. Additionally, the definitions of “young” and “typical-onset” patients are historically divided at 50 years of age; we considered that there might be differences that would be more apparent if those closest to 50 were considered as a distinct group.

This study aimed to identify differences between age cohorts, independent of demographics, to elucidate important distinctions in both tumor biology and oncologic management. We hypothesized that patients under the age of 40 would be distinctly different from patients aged 40 to 49 and over 50 years with respect to demographics, tumor characteristics, and treatment. We also predicted differences in tumor biology and treatment would persist despite controlling for demographic differences.

## Materials and methods

The National Cancer Database (NCDB) was queried for rectal adenocarcinoma patients from 2004 to 2014. The NCDB is a large, facility-based, oncologic data set that currently captures 70% of all newly diagnosed malignancies in the United States. This database serves as a representative sample of oncologic care across the US and was therefore utilized to minimize selection bias and optimize sample size. The University Hospitals Cleveland Medical Center Institutional Review Board (IRB) designated this study as non-human subjects research and was therefore exempt from IRB approval. 

Definitions

Rectal cancer patients with adenocarcinoma, including those with mucinous, signet ring cell, and non-mucinous adenocarcinoma histology, were included. Patients missing stage and treatment data were excluded from the analysis. Patients who received radiation to sites other than the pelvis were also excluded, as evidence of treatment for metastatic disease or other primary cancers could not be verified. Patients were grouped by age at diagnosis using commonly accepted nomenclature, and age ranges from the literature, with early-onset (EO) defined as < 40 years of age and typical-onset (TO) > 50 years of age. A third range, mid-age onset (MO) 40 to 49, was created to further delineate young from typical-onset and isolate differences.

Statistical analysis

Univariate analysis was performed with a pairwise Chi-square test with Bonferroni correction for all categorical data. Variables of interest included age, gender, race, median income, urbanization classification, insurance, Charlson/Deyo score, AJCC clinical stage, histology, grade, chemotherapeutic usage and timing, radiation dose and timing, and surgical intervention. All the demographic and clinical variables are presented in tables with frequency (%). Missing data were excluded from analysis and marked as such within the tables unless noted within the NCDB as unknown. 

To control for the potential confounding effects of patient demographics on both tumor features and treatment modalities, a propensity score match was performed using demographic covariates found to be statistically different between the groups. Variables included in the propensity match analysis were gender, race, urbanization classification, income, insurance status, and Charlson/Deyo score. Patients with missing data on propensity-matched variables were excluded from the analysis. Nearest neighbor 1:1 matching without replacement was used, with caliper set to 0.001. The area of common support was verified, and the balance between the resultant matched cohorts was confirmed with a bivariate analysis of the covariates. Separate propensity match analyses were performed between EO and TO groups and EO and MO groups. Statistical analysis was performed with Stata 16 (StataCorp, College Station, Texas) and SAS (SAS Institute, Cary, North Carolina). 

## Results

Patient demographics

**Figure 1:**
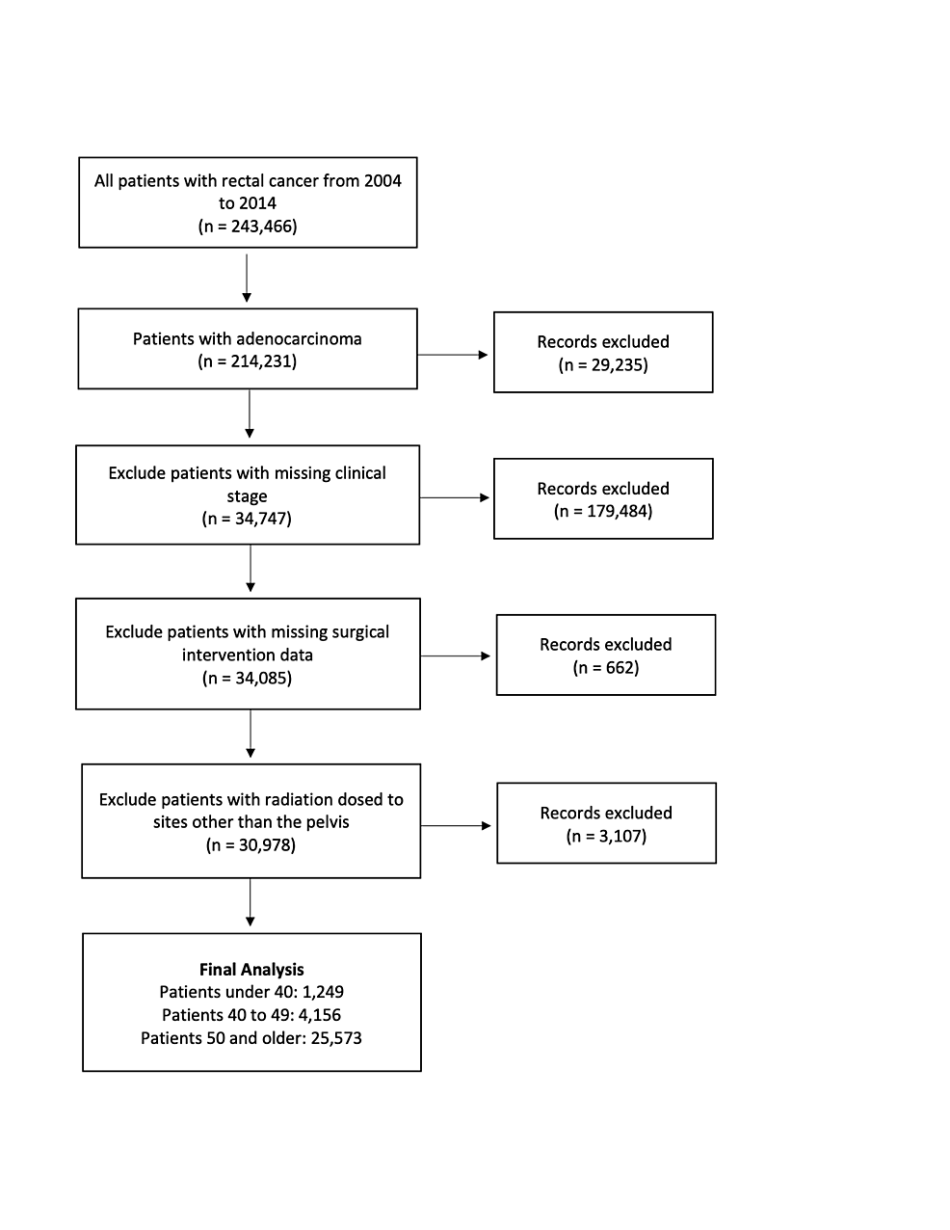
Inclusion and exclusion criteria algorithm

Following exclusion criteria, 30,978 patients were included in this study: 1,249 EO, 4,156 MO and 25,573 TO (Figure 1). The majority of patients were male (60.1%), white (87.1%), living in metropolitan locations (79.4%), and with private insurance (48.1%). The most common presentation was non-mucinous adenocarcinoma (93.3%), clinical-stage II disease (29.7%), and moderate differentiation (74.8%). 

Pairwise analysis of patient characteristics across the three age groups demonstrated significant differences in nearly all variables, as shown in Table 1. EO and MO groups were similar only with respect to race and urbanization classification, containing similar proportions of non-white patients and patients living in metropolitan areas, while TO had significantly larger numbers of white patients (EO 83.0%, MO 83.8%, TO 87.9%) and were least likely to live in metropolitan locations (EO 83.8%, MO 81.2%, TO 78.9%). The remainder of the variables of interest were significantly different between all three groups. Females were more prevalent among the younger cohorts (EO 47.7%, MO 42.0%, TO 39.1%, P<0.001), as were patients with Spanish/Hispanic ancestry (EO 9.2%, MO 5.9%, TO 4.6%, P<0.001). MO contained the highest percentage of patients with private insurance, followed by EO and TO (EO 75.3%, MO 78.0%, TO 42.0%, P<0.001), while TO had the greatest number of patients on Medicare (EO 1.76%, MO 4.0%, TO 48.6%, P<0.001). The MO group contained the greatest percentage of patients with incomes in the highest quartile, compared to EO and TO (EO 34.3%, MO 35.0%, TO 31.3%, P<0.017). Charlson/Deyo score (CD) also differed across groups, with comorbidity rising with age (CD score of 2, EO 0.4%, MO 1.8%, TO 5.1%, P<0.001).

**Table 1:** Patient demographics and tumor characteristics with a pairwise comparison between all three age groups (n = 30,978) *Due to Bonferroni correction, significance was set at P < 0.017. EO - early-onset, MO - mid-age onset, TO - typical-onset, AJCC - American Joint Committee on Cancer

	Age group			Pairwise Chi-square P values			
	EO n (%) n=1,249	MO n (%) n=4,156	TO n (%) n=25,573	EO to MO		MO to TO	EO to TO
Sex							
Male	653 (52.28)	2409 (57.96)	15564 (60.86)	<0.001		<0.001	<0.001
Female	596 (47.72)	1747 (42.04)	10009 (39.14)				
Race							
White	1037 (83.03)	3482 (83.78)	22476 (87.89)	0.020		<0.001	<0.001
Black	112 (8.96)	426 (10.25)	1815 (7.10))				
Other	100 (8.01)	248 (5.97)	1282 (5.01)				
Spanish/Hispanic ancestry							
None	1045 (83.67)	3585 (86.26)	22457 (87.82)	<0.001		<0.001	<0.001
Yes	115 (9.21)	247 (5.94)	1169 (4.57)				
Unknown	89 (7.12)	324 (7.80)	1947 (7.61)				
Urbanization classification							
Metropolitan	1046 (83.75%)	3376 (81.23)	20179 (78.91)	0.160		0.004	<0.001
Urban	139 (11.13)	557 (13.40)	3939 (15.40)				
Rural	20 (1.60)	80 (1.92)	570 (2.23)				
Unknown	44 (3.52)	143 (3.44)	885 (3.46)				
Median income by ZIP Code							
< $38,000	167 (13.54)	623 (15.23)	4297 (17.08)	0.005		<0.001	<0.001
$38,000 to $47,999	320 (25.95)	870 (21.27)	6117 (24.32)				
$48,000 to $62,999	323 (26.20)	1166 (28.50)	6858 (27.26)				
≥ $63,000	423 (34.31)	1432 (35.00)	7882 (31.33)				
Insurance status							
None	96 (7.69)	256 (6.16)	726 (2.84)	<0.001		<0.001	<0.001
Private	941 (75.34)	3240 (77.96)	10733 (41.97)				
Medicaid	147 (11.77)	374 (9.00)	1054 (4.12)				
Medicare	22 (1.76)	165 (3.97)	12422 (48.57)				
Other government	15 (1.20)	50 (1.20)	263 (1.03)				
Unknown	28 (2.24)	71 (1.71)	375 (1.47)				
Charlson-Deyo score							
0	1158 (92.71)	3745 (90.11)	19459 (76.09)	<0.001		<0.001	<0.001
1	86 (6.89)	337 (8.11)	4811 (18.81)				
2	5 (0.40)	74 (1.78)	1303 (5.10)				
AJCC clinical stage							
0	32 (2.56)	151 (3.63)	1525 (5.96)	<0.001		<0.001	<0.001
I	200 (16.01)	843 (20.28)	7025 (27.47)				
II	298 (23.86)	1121 (26.97)	7766 (30.37)				
III	540 (43.23)	1486 (35.76)	7016 (27.44)				
IV	179 (14.33)	555 (13.35)	2241 (8.76)				
Histology							
Adenocarcinoma	1119 (89.59)	3849 (92.61)	23933 (93.59)	<0.001		0.003	<0.001
Mucinous	100 (8.01)	263 (6.33)	1479 (5.78)				
Signet ring cell	30 (2.40)	44 (1.06)	161 (0.63)				
Grade							
Well	89 (8.29)	287 (7.95)	2171 (9.78)	0.002		0.014	<0.001
Moderate	761 (70.86)	2744 (75.99)	16613 (74.83)				
Poor	202 (18.81)	541 (14.98)	3195 (14.39)				
Anaplastic	22 (2.05)	39 (1.08)	221 (1.00)				
Indeterminate	175 (14.01)	545 (13.11)	3,373 (13.19)				

Analysis of tumor factors 

As shown in Table 1, there are significant differences in tumor characteristics between all three groups on pairwise analysis. The clinical stage at the time of diagnosis rose as age decreased, with 57.5% EO, 49.2% MO, and 36.2% TO presenting with a clinical stage of III or higher (P<0.017). Non-mucinous adenocarcinoma was the most prevalent pathology among all groups (EO 89.6%, MO 92.6%, TO 93.6%, P<0.017), with mucinous and signet ring cell histology proportionally higher in EO and MO groups. Moderate differentiation was the most common histologic grade among all three groups (EO 60.9%, MO 66.0%, TO 65.0%, P<0.017), with the EO group containing higher percentages of poorly differentiated and anaplastic tumors compared to MO and TO. Of note, analysis of additional biological features, such as microsatellite instability, KRAS mutations, lymphovascular invasion, and perineural invasion were not included in this analysis due to limited data. 

Analysis of treatment modalities

Analysis of treatment modalities and tumor response demonstrated significant differences between all three groups with regards to treatment, as demonstrated in Table 2. 

**Table 2:** Oncologic management of patients by age group, all stages (n=30,978) Due to Bonferroni correction, all significant values are P < 0.017. Variables with missing data have been denoted (*) and statistical analysis has been performed to exclude missing values. EO - early-onset, MO - mid-age onset, TO - typical-onset

	Age group			Pairwise Chi-square P values		
	EO n(%) n=1,249	MO n(%) n=4,156	TO n(%) n=25,573	EO to MO	MO to TO	EO to TO
Chemotherapy*						
Not given	169 (13.66)	720 (17.56)	8351 (33.15)	0.001	<0.001	<0.001
Given (number of agents unknown)	108 (8.73)	313 (7.63)	1923 (7.63)			
Single-agent chemotherapy	368 (29.75)	1328 (32.38)	8311 (33.00)			
Multi-agent chemotherapy	592 (47.86)	1740 (42.43)	6603 (26.21)			
Chemotherapy timing*						
None	135 (13.76)	606 (18.84)	6659 (34.52)	0.001	<0.001	<0.001
Neoadjuvant only	394 (40.16)	1311 (40.76)	7082 (36.71)			
Adjuvant only	166 (16.92)	516 (16.04)	2915 (15.11)			
Neoadjuvant & adjuvant	286 (29.15)	783 (24.35)	2637 (13.67)			
Radiation timing*						
None	271 (21.93)	1022 (24.79)	9850 (38.97)	0.019	<0.001	<0.001
Neoadjuvant only	818 (66.18)	2550 (61.86)	12529 (49.56)			
Adjuvant only	136 (11.00)	526 (12.76)	2813 (11.13)			
Neoadjuvant & adjuvant	11 (0.89)	24 (0.58)	86 (0.34)			
Radiation dosing						
None	271 (21.70)	1022 (24.59)	9862 (38.56)	0.176	<0.001	<0.001
< 45 Gray	44 (3.52)	137 (3.30)	933 (3.65)			
45 to 50 Gray	664 (53.16)	2166 (52.12)	10737 (41.99)			
> 50 Gray	270 (21.62)	831 (20.00)	4041 (15.8)			
Surgical intervention						
None	16 (1.28)	72 (1.73)	556 (2.17)	0.224	<0.001	<0.001
Local tumor destruction	0 (0)	1 (0.02)	9 (0.04)			
Local tumor excision	54 (4.32)	229 (5.51)	2482 (9.71)			
Surgical resection	1179 (94.4)	3854 (92.73)	22526 (88.09)			

EO patients were significantly more likely to receive multi-agent chemotherapy than older patients (EO 47.9%, MO 42.4% and TO 26.2%, P<0.001), with EO and MO patients more frequently receiving neoadjuvant and adjuvant dosing than the TO group. Neoadjuvant radiation therapy was dosed most frequently among the EO group (EO 66.1%, MO 61.8%, and TO 49.5%, P<0.017). Total radiation dosing was similar between EO and MO groups, with these patients more frequently receiving > 50.4 Gy (21.6% EO and 20.0% MO) than the TO group (15.8% TO). Type of surgical intervention was also similar between EO and MO groups, more often undergoing radical surgical resection (94.4% EO, 92.7% MO, 88.1% TO), compared to the higher rate of local tumor excision among TO patients (4.3% EO, 5.5% MO, 9.7% TO).

Propensity matched cohorts: EO vs. MO

Propensity matched cohorts were created between EO and MO groups based on gender, race, geographic location, income, Charlson/Deyo score, and insurance status, resulting in 1,173 patients within each cohort (Table 3). No significant difference was found among matched variables in univariate analysis. The EO cohort contained significantly more clinical stage III and IV patients (43.7% and 14.5%, respectively) when compared to the MO cohort (35.6% and 13.8%, respectively). Grade and histology were not significantly different, however, with EO and MO groups containing similar proportions of non-mucinous adenocarcinoma (89.4% EO and 92.1% MO) with moderate differentiation (61.4% EO and 66.5% MO). Administration of radiation and chemotherapy was also similar across EO and MO propensity-matched groups when stratified by stage (Table 4). A significant difference was only found amongst stage III patients, with 52.2% of the EO group receiving multi-agent chemotherapy, compared to 43.8% of the MO group. 

**Table 3:** Clinicodemographic characteristics of propensity matched age cohorts (EO/MO n=2,346; EO/TO n=2,400) Significant values are P < 0.05. EO - early-onset, MO - mid-age onset, TO - typical-onset, AJCC - American Joint Committee on Cancer

	EO n(%) n=1,173	MO n(%) n=1,173	P Value	EO n(%) n=1,200	TO n(%) n=1,200	P value
Sex						
Male	621 (52.94%)	619 (52.77%)	0.934	630 (52.5)	632 (52.67)	0.935
Female	552 (47.06%)	554 (47.23%)		570 (47.5)	568 (47.33)	
Race						
White	981 (83.63%)	993 (84.65%)	0.951	999 (83.25)	1001 (83.42)	0.780
Black	109 (9.29%)	99 (8.44%)		109 (9.08)	103 (8.58)	
Other	83 (7.08)	81 (6.91)		92 (7.66)	96 (8.00)	
Spanish/Hispanic ancestry						
None	995 (84.83%)	1011 (86.19%)	0.636	1005 (83.75)	1005 (83.75)	0.954
Unknown	85 (7.246%)	76 (6.479%)		85 (7.08)	88 (7.33)	
Yes	93 (7.928%)	86 (7.332%)		110 (9.17)	107 (8.92)	
Urbanization classification						
Metropolitan	1015 (86.53%)	1017 (86.701%)	0.948	1041 (86.75)	1044 (87.00)	0.982
Urban	138 (11.77%)	138 (11.765%)		139 (11.58)	136 (11.33)	
Rural	20 (1.71%)	18 (1.535%)		20 (1.67)	20 (1.67)	
Median income (by ZIP Code)						
< $38,000	163 (13.90)	163 (13.90)	0.985	315 (26.25)	312 (26)	0.998
38,000 to 47,999	294 (25.06)	298 (25.41)		320 (26.67)	320 (26.67)	
48,000 to 62,999	320 (27.28)	312 (26.60)		404 (33.67)	404 (33.67)	
≥ 63,000	396 (33.76)	400 (34.10)		164 (13.67)	164 (13.67)	
Insurance type						
None	85 (7.25)	93 (7.93)	0.922	92 (7.67)	92 (7.67)	0.970
Private	895 (76.30)	888 (75.70)		902 (75.17)	908 (75.67)	
Medicaid	139 (11.85)	142 (12.11)		145 (12.08)	147 (12.25)	
Medicare	18 (1.54)	21 (1.79)		20 (1.67)	20 (1.67)	
Other government	13 (1.11)	10 (0.85)		14 (1.17)	11 (0.92)	
Unknown	23 (1.96)	19 (1.62)		27 (2.25)	22 (1.83)	
Charlson-Deyo score						
0	1088 (92.75)	1099 (93.70)	0.648	1112 (92.67)	1111 (92.58)	0.997
1	80 (6.82)	69 (5.88)		83 (6.92)	84 (7.00)	
2	5 (0.43)	5 (0.43)		5 (0.42)	5 (0.42)	
AJCC clinical stage						
0	31 (2.64)	44 (3.75)	<0.001	31 (2.58)	72 (6.00)	<0.001
I	182 (15.52)	231 (19.69)		190 (15.83)	319 (26.58)	
II	277 (23.62)	318 (27.11)		283 (23.58)	336 (28.00)	
III	513 (43.73)	418 (35.64)		521 (43)	375 (31.25)	
IV	170 (14.49)	162 (13.81)		175 (14.58)	98 (8.17)	
Histology						
Adenocarcinoma	1049 (89.43)	1080 (92.07)	0.055	1074 (89.5)	1128 (94)	<0.001
Mucinous	96 (8.18)	77 (6.56)		98 (8.17)	63 (5.25)	
Signet ring cell	28 (2.39)	16 (1.36)		28 (2.33)	9 (0.75)	
Grade						
Well differentiated	79 (6.74)	76 (6.48)	0.091	82 (6.83)	98 (8.17)	0.195
Moderately differentiated	720 (61.38)	780 (66.50)		734 (61.17)	756 (63)	
Poorly differentiated	182 (15.52)	155 (13.21)		192 (16.00)	160 (13.33)	
Anaplastic	21 (1.79)	13 (1.11)		21 (1.75)	14 (1.17)	
Indeterminate	171 (14.58)	149 (12.70)		171 (14.25)	172 (14.33)	

**Table 4:** Oncologic management among propensity-matched groups, stratified by clinical stage Significant values are P < 0.05. Variables with missing data have been denoted (*) and statistical analysis has been performed to exclude missing values. EO - early-onset, MO - mid-age onset, TO - typical-onset

Propensity-matched EO to MO									
	Stage II EO n(%) n=277	Stage II MO n(%) n=318	P Value	Stage III EO n(%) n=513	Stage III MO n(%) n=418	P Value	Stage IV EO n(%) n=170	Stage IV MO n(%) n=162	P value
Radiation timing*									
Adjuvant only	31 (11.31)	45 (14.24)	0.726	57 (11.22)	54 (13.08)	0.755	13 (7.83)	10 (6.25)	0.574
Neoadjuvant only	211 (77.01)	234 (74.05)		415 (81.69)	334 (80.87)		91 (54.82)	80 (50.00)	
Neoadjuvant & Adjuvant	3 (1.09)	4 (1.27)		5 (0.98)	3 (0.72)		2 (1.2)	4 (2.50)	
None	29 (10.58)	33 (10.44)		31 (6.11)	22 (5.33)		60 (36.14)	66 (41.25)	
Radiation dose									
<45 Gray	8 (2.89)	5 (1.57)	0.348	13 (2.53)	12 (2.87)	0.884	16 (9.41)	6 (3.70)	0.143
45 to 50 Gray	180 (64.98)	194 (61.01)		329 (64.13)	276 (66.03)		65 (38.24)	57 (35.19)	
> 50.4 Gray	60 (21.66)	86 (27.04)		140 (27.29)	108 (25.84)		29 (17.06)	33 (20.37)	
None	29 (10.47)	33 (10.38)		31 (6.04)	22 (5.26)		60 (35.29)	66 (40.74)	
Chemotherapy*									
Given (number of agents unknown)	34 (12.45)	25 (7.96)	0.165	50 (9.75)	44 (10.58)	0.048	5 (2.99)	10 (6.29)	0.140
Single-agent chemotherapy	107 (39.19)	144 (45.86)		183 (35.67)	173 (41.59)		23 (13.77)	23 (14.47)	
Multi-agent chemotherapy	115 (42.12)	122 (38.85)		268 (52.24)	182 (43.75)		135 (80.84)	116 (72.96)	
Not given	17 (6.23)	23 (7.32)		12 (2.34)	17 (4.09)		4 (2.40)	10 (6.29)	
Chemotherapy timing*									
Adjuvant only	31 (14.55)	42 (16.67)	0.901	61 (14.99)	40 (12.35)	0.137	34 (23.61)	26 (18.84)	0.356
Neoadjuvant only	99 (46.48)	115 (45.63)		192 (47.17)	163 (50.31)		55 (38.19)	50 (36.23)	
Neoadjuvant & adjuvant	66 (30.99)	73 (28.97)		142 (34.89)	102 (31.48)		48 (33.33)	47 (34.06)	
None	17 (7.98)	22 (8.73)		12 (2.95)	19 (5.86)		7 (4.86)	15 (10.87)	
Propensity-matched EO to TO									
	Stage II EO n(%) n=283	Stage II TO n(%) n=336	P Value	Stage III EO n(%) n=521	Stage III TO n(%) n=375	P Value	Stage IV EO n(%) n=175	Stage IV TO n(%) n=98	P value
Radiation timing*									
Adjuvant only	32 (11.43)	44 (13.33)	0.794	58 (11.24)	46 (12.53)	0.013	14 (8.19)	9 (9.38)	0.035
Neoadjuvant only	215 (76.79)	246 (74.55)		421 (81.59)	273 (74.39)		94 (54.97)	37 (38.54)	
Neoadjuvant & adjuvant	3 (1.07)	2 (0.61)		5 (0.97)	3 (0.82)		2 (1.17)	0	
None	30 (10.71)	38 (11.52)		32 (6.20)	45 (12.26)		61 (35.67)	50 (52.08)	
Radiation dose									
<45 Gray	8 (2.83)	9 (2.68)	0.840	13 (2.50)	9 (2.40)	0.015	16 (9.14)	7 (7.14)	0.037
45 to 50 Gray	183 (64.66)	206 (61.31)		332 (63.72)	234 (62.40)		69 (39.43)	24 (24.49)	
> 50.4 Gray	62 (21.91)	82 (24.40)		144 (27.64)	87 (23.2)		29 (16.57)	17 (17.35)	
None	30 (10.60)	39 (11.61)		32 (6.14)	45 (12.00)		61 (34.86)	50 (51.02)	
Chemotherapy*									
Given (number of agents unknown)	36 (12.90)	32 (9.58)	0.002	50 (9.60)	33 (8.82)	0.001	5 (2.91)	8 (8.42)	0.022
Single-agent chemotherapy	108 (38.71)	166 (49.70)		188 (36.08)	145 (38.77)		25 (14.53)	17 (17.89)	
Multi-agent chemotherapy	117 (41.94)	100 (29.94)		270 (51.82)	167 (44.65)		138 (80.23)	63 (66.32)	
Not given	18 (6.45)	36 (10.78)		13 (2.50)	29 (7.75)		4 (2.32)	7 (7.36)	
Chemotherapy timing*									
Adjuvant only	32 (14.75)	25 (10.29)	0.020	61 (14.73)	49 (16.5)	<0.001	36 (24.32)	29 (32.95)	0.004
Neoadjuvant only	100 (46.08)	132 (54.32)		197 (47.58)	130 (43.77)		56 (37.84)	31 (35.23)	
Neoadjuvant & adjuvant	67 (30.88)	53 (21.81)		144 (34.78)	87 (29.29)		49 (33.11)	15 (17.05)	
None	18 (8.29)	33 (13.58)		12 (2.90)	31 (10.44)		7 (4.73)	13 (14.77)	

Propensity-matched cohorts: EO vs. TO

Propensity-matched cohorts created between EO and TO groups, based upon the patient traits previously listed, resulted in 1,200 patients per group (Table 3). No significant differences were noted in matched variables in univariate analysis. Clinical stage III and IV disease was significantly higher in the EO group (43.0% stage III and 14.6% stage IV) compared to the TO group (31.3% stage III and 8.2% stage IV), P<0.001. The prevalence of non-mucinous adenocarcinoma was significantly lower in the EO group when compared to the TO group (89.5% EO and 94.0% TO, P<0.001). No difference was noted in proportion of histologic grade, with moderate histology remaining the most common presentation (61.2% EO and 63.0% TO). Administration of chemotherapy and radiation was significantly different between EO and TO propensity-matched groups, particularly among stage III and stage IV patients. As Table 4 demonstrates, stage III and IV EO patients were significantly more likely than the TO propensity-matched group to receive neoadjuvant radiation at or above NCCN guideline dosing, with multi-agent chemotherapy, dosed both in the neoadjuvant and adjuvant setting (P<0.05).

## Discussion

This study utilized a large national database to evaluate age-related differences in patient demographics, tumor characteristics, and treatment patterns among patients with rectal adenocarcinoma. It appears that there are important demographic differences within the group of patients historically considered early age of onset, as well as those at a typical age of onset. However, once these demographics are accounted for, tumor characteristics become more similar. However, oncologic treatment patterns differ: following control of demographics, a stage-matched analysis of oncologic management demonstrated significant differences between age groups, with young patients more likely to receive radiation and chemotherapy.

Prior literature has thoroughly examined the demographic and clinicopathologic differences among patients with CRC, determining that patients under the age of 50 more frequently come from minority backgrounds, have more advanced disease, and worse histopathological features [9-11]. It has been theorized that young-onset CRC, given their demographic and pathologic differences, may represent a unique disease process [12]. However, clinicodemographic data from rectal cancer patients, exclusive of colon cancer, have not been thoroughly investigated. One study of the Surveillance, Epidemiology, and End Results Program (SEER) database revealed that young rectal cancer patients were more likely to be minorities, have poorly differentiated tumors, and have advanced disease [13]. Our analysis of a separate national database supports these prior findings and adds to the existing literature, which suggests that clinicodemographic disparities seen in CRC overall remain valid within the rectal cancer population, even as the rates of rectal and left-sided colon cancer in young patients increase disproportionately to right-sided colon cancer [13,14]. In addition, this analysis demonstrates that not all patients under the age of 50 should be regarded as one cohort, given the presence of a significant degree of clinicodemographic variation between EO and MO groups; when considering those under age 40, the differences are even more apparent. 

To better examine the relationship between age and tumor features, this study evaluated differences in tumor characteristics between age cohorts after controlling for demographic variation. Tumor characteristics became more similar among age groups, with two exceptions: clinical stage at presentation and tumor histology. The reasons for the differences in the clinical stage are only partially explained by age-based screening, which has previously been described [15]. An analysis of nearly 500 CRC patients from a single institution revealed patients under 50 years of age had significantly longer median time to diagnosis, symptom duration, and time to evaluation than their older counterparts [16]. In the current study, both EO and MO groups were outside of screening guidelines, yet the EO group still presented at higher stages than the MO group. This suggests that screening guidelines are not the sole driver of delayed diagnosis and advanced stage at presentation. Additional factors that may lead to delays in diagnosis in younger patients may be related to both patient and clinician awareness of the significance of symptoms in this age group. Higher clinical stage at the time of diagnosis in EO groups may also be related to the histologic differences observed. 

With regard to the differences in histology after controlling for demographics, mucinous and signet ring adenocarcinomas remained significantly more prevalent among EO patients compared to matched TO patients, but not among the matched MO and TO cohorts. This is consistent with findings in other studies, which have demonstrated that early-onset CRC patients have higher rates of signet ring and mucinous tumor histology [4,17]. One study evaluated 333 rectal cancer patients under age 50, matched with 675 rectal cancer patients over age 65, finding that the younger group mirrored the older cohort in terms of demographics and tumor characteristics, such as histologic grade and tumor size, yet presented more frequently with advanced stage and mucinous or signet ring histology [14]. These differences may be related to tumor biology, as genetic analysis has demonstrated differences in gene expression and regulation, with the mitogen-activated protein kinase (MAPK) signaling pathway disproportionately affected in young patients [18].

Screening guidelines are predominantly based on age, and more recently, have included the Black race. This analysis, in the context of prior literature, suggests patient demographics other than age may drive a significant amount of the variability in tumor characteristics that have been noted between young and typical-onset patient populations. As a result, screening and treatment guidelines based upon age may not be sufficient and are therefore likely to miss younger patients at greatest risk for disease. As such, there is a need to identify additional risk factors for younger patients and include them in screening guidelines. Demographic factors, such as race and Hispanic ethnicity, as demonstrated by this analysis, may play a role in risk for early-onset rectal cancer. Environmental exposures, such as western, low-fiber diets, and obesity, have also been tied to increased risk for rectal cancer and should also be evaluated for inclusion in screening criteria [19].

Because age is not a good proxy for tumor behavior, tumor characteristics, rather than demographics, should inform oncologic management. However, this study identified that stage-specific oncologic management was strikingly different between young and typical-onset patients following propensity matching, with the younger patients of the EO and MO groups receiving more chemotherapy and radiation than TO patients. This suggests that providers delivered care to EO and MO groups more uniformly while TO patients received different management. Furthermore, the propensity-matched analysis performed in this study included the Charlson/Deyo score, a weighted score of comorbidities reported within the NCDB, and therefore controls for the effect of comorbidity on oncologic management. Since most patients had a Charlson/Deyo score of 0 or 1 in the TO group, treatment differences cannot be explained by increased rates of comorbidities. Rather, this difference in treatment suggests an age bias unrelated to comorbidity, insurance status, or other demographics. Prior literature supports these findings, as young patients have been documented to more frequently receive radiation, chemotherapy, and sphincter-sparing surgical resections than older patients [14,20].

Limitations

This study is limited by the nature of administrative databases, such as the NCDB. The depth of this analysis was limited by a lack of data granularity, such as the precise chemotherapy regimen. Furthermore, data on microsatellite instability, Kirsten rat sarcoma virus (KRAS) mutations, and other prognostic indicators, such as perineural invasion, were frequently missing. The limited number of patients with these data points led us to remove these variables from analysis, which restricted the investigation of tumor characteristics. Given these restrictions of the NCDB dataset, there may be differences in tumor biology that remained undetected by our analysis. Data missing within the NCDB, if not labeled as "unknown" by the NCDB dataset, were assumed to be missing at random. The principle of pairwise deletion was applied to account for any missing data within variables of interest. 

Future Directions

Differences seen in tumor characteristics among early-onset and typical-onset rectal cancer patient cohorts are related to demographic differences other than age at diagnosis. Given these findings, in context with known data on biological differences between tumors of typical-onset and younger patients, early-onset rectal cancers are likely to be the product of a complex interplay between demographics, genetic predisposition, and as-of-yet unknown contributing environmental exposures, such as diet or an individual's microbiome. With increasing evidence of different tumor biology based on demographics, there is a need to develop alternative screening and treatment algorithms inclusive of demographic risk factors other than age. Future research is needed to evaluate rectal cancer characteristics among early-onset patients with greater regard for the impact of patient demographics on tumor biology and response to treatment. Additionally, further investigation is needed to evaluate reasons for age-related variation in the management of rectal cancer patients.

## Conclusions

This analysis demonstrated significant clinicodemographic differences between age cohorts in a large national database of rectal cancer patients. However, only differences in clinical stage, histology, and oncologic management remained following control of patient demographics. This suggests that early-onset patients may be subject to different oncologic processes and tumor biology-related demographic influences, such as geographic location, gender, or race, rather than age alone. Furthermore, younger patients more often receive multi-agent neoadjuvant chemotherapy and radiation when tumor and patient characteristics are comparable. Reasons for age-related variation in management, along with additional investigation of tumor biology among younger patients are areas of future study. 
